# Pernicious Anemia: Basic Pathophysiology and Diagnostic Challenges in Neuropsychiatric Patients

**DOI:** 10.3390/hematolrep18040047

**Published:** 2026-07-01

**Authors:** Yin Mon Myat, Kyaw Zin Thein, Thein Hlaing Oo

**Affiliations:** 1 Interfaith Medical Center, Brooklyn, NY 11213, USA; yinmon.myat@obhny.org; 2 Comprehensive Cancer Centers of Nevada, Las Vegas, NV 89128, USA; kyaw.thein@usoncology.com; 3 Kirk Kerkorian School of Medicine at University of Nevada, Las Vegas (UNLV), Las Vegas, NV 89106, USA; 4 Department of Medicine, Touro University Nevada, Henderson, NV 89014, USA; 5 Section of Hematology, The University of Texas M.D. Anderson Cancer Center, Houston, TX 77030, USA

**Keywords:** pernicious anemia, diagnostic difficulties, cobalamin deficiency, chronic atrophic gastritis, intrinsic factor antibody, parietal cell antibody, neuropsychiatric patients, homocysteine, methylmalonic acid

## Abstract

Pernicious anemia (PA) represents a significant diagnostic challenge in neuropsychiatric patients due to its subtle and variable presentation. While PA is traditionally associated with clinical and biochemical manifestations of anemia, many patients, particularly those with neuropsychiatric symptoms, may have normal hematologic parameters, delaying recognition and appropriate treatment. Neurological and psychiatric symptoms, ranging from cognitive impairment and mood disorders to subacute combined degeneration (SCD) of the spinal cord, can precede hematologic abnormalities, leading to misdiagnosis or inappropriate management. The lack of a definitive gold standard test for cobalamin deficiency (CD) further complicates identification. Commonly used biomarkers, such as serum cobalamin, methylmalonic acid (MMA), homocysteine (Hcy), intrinsic factor antibodies (IFAs), and parietal cell antibodies (PCAs), each have limitations in diagnosing PA, especially in the absence of overt anemia. The variability in diagnostic criteria and cutoff values across studies adds to the challenge of achieving early and accurate diagnosis. This article reviews the complexities of diagnosing PA in neuropsychiatric patients, evaluates the limitations of current diagnostic methods, and emphasizes the need for a more comprehensive, standardized approach to early detection and treatment. Combining clinical awareness with improved biomarker interpretation is essential for preventing irreversible neurological damage and improving patient outcomes. Improved diagnostic protocols and further research are essential to optimize detection and minimize the risk of long-term neurological damage.

## 1. Introduction

Pernicious anemia (PA), a known complication of autoimmune chronic atrophic gastritis (ACAG), is a leading cause of severe cobalamin deficiency (CD). It results from lack of intrinsic factor (IF), which impairs cobalamin absorption in the terminal ileum. IF deficiency is primarily caused by IF antibodies (IFAs). PA affects approximately 0.1% of the general population, with an incidence of 2–3% in those over the age of 65, though true prevalence may be underestimated due to asymptomatic cases [1]. Diagnosis is based on clinical presentation and laboratory findings of anemia and CD. However, in neuropsychiatric patients, overlapping symptoms complicate recognition, often leading to delays. Moreover, multiple biomarkers must be carefully evaluated to confirm PA.

Although numerous reviews have examined varying presentations of CD, PA represents a distinct autoimmune etiology with unique diagnostic considerations. In addition, the majority of the existing literature focuses on the hematological manifestations of PA despite evidence that neuropsychiatric symptoms may precede anemia or macrocytosis, contributing to misdiagnosis and delays in treatment [2]. While the neuropsychiatric manifestations are attributable to CD regardless of etiology, the autoimmune nature of PA introduces additional diagnostic challenges. Variability in diagnostic methods, biomarker performance, and the lack of standardized diagnostic thresholds further complicate diagnosis. This review provides a focused overview of the pathophysiology of PA, highlights neuropsychiatric presentations and common misdiagnosis scenarios, critically evaluates the strengths and limitations of current diagnostic biomarkers, and discusses practical approaches to overcoming diagnostic pitfalls in clinical practice.

## 2. Basic Pathophysiology of Pernicious Anemia

**Figure 1:**
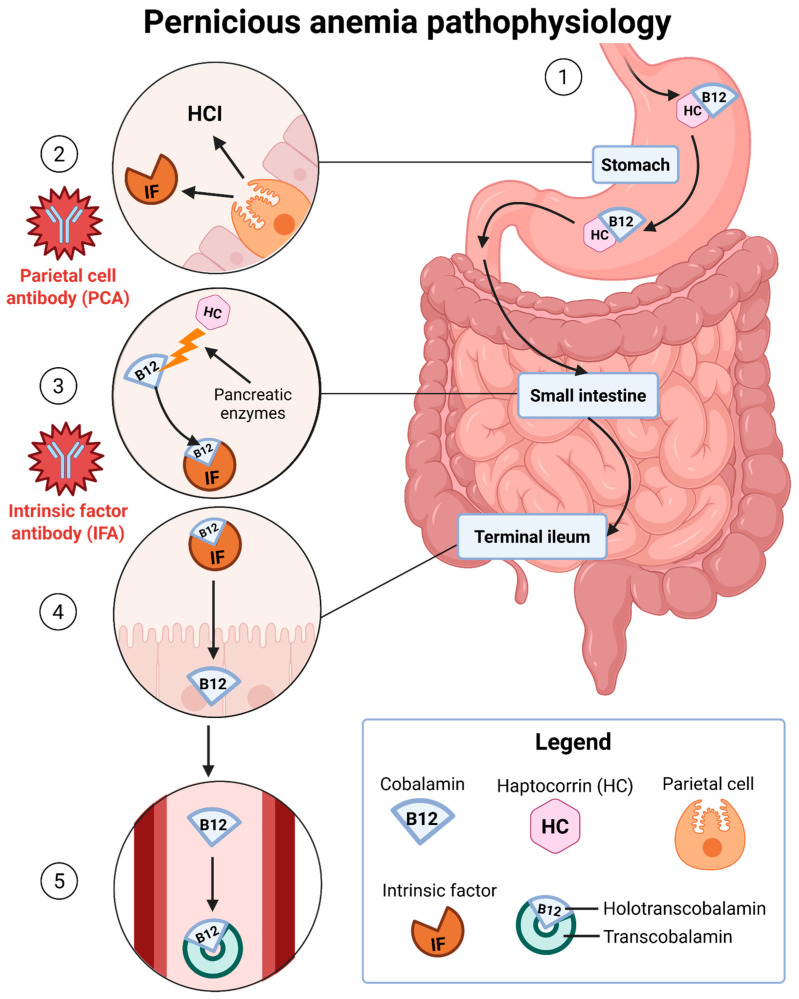
Pathophysiological pathway of physiologic cobalamin absorption and autoimmune disruption. (1) Gastric binding of free cobalamin to haptocorrin (HC); (2) Parietal cell secretion of hydrochloric acid (HCI) and intrinsic factor (IF); (3) Pancreatic enzyme degradation of the HC–cobalamin complex in the duodenum, enabling free cobalamin to bind IF; (4) Receptor-mediated absorption of the IF–cobalamin complex in the terminal ileum, followed by peripheral tissue transport via transcobalamin II (TCII) as holotranscobalamin (holoTC). In pernicious anemia, autoimmune destruction mediated by parietal cell antibodies (PCAs) and intrinsic factor antibodies (IFAs) interrupts this transport cascade, precipitating systemic cobalamin deficiency. Created in BioRender. Myat, Y. (2026) https://BioRender.com/1zwhflt, accessed 18 June 2026.

**Figure 2:**
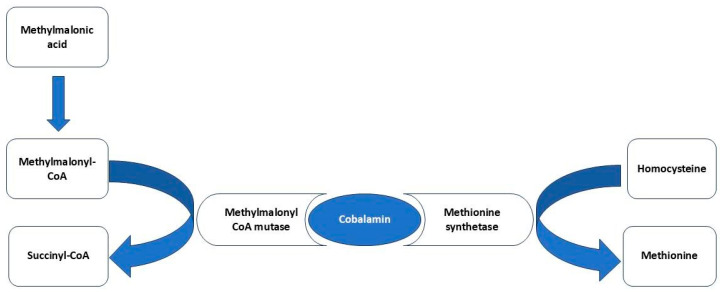
Cobalamin cofactor for methionine synthetase and methylmalonyl CoA mutase and its deficiency leading to high levels of homocysteine and methylmalonic acid.

Understanding the pathophysiology of PA is essential for recognizing its clinical manifestations and diagnostic challenges. Dietary cobalamin binds to salivary haptocorrin (HC), also known as R-protein or transcobalamin I, to form a complex. The majority of cobalamin–HC complexes are broken down by peptide-mediated proteolysis and by the gastric acid. Pancreatic enzymes also cause further breakdown of the complex. The IF produced by gastric parietal cells (PCs) binds to the free cobalamin molecule. The IF-cobalamin complex travels to the brush border of the terminal ileum. It binds to the cubilin receptor whereby the process of endocytosis occurs and IF is destroyed. Cobalamin is absorbed into the bloodstream where it is bound to either HC or transcobalamin II (TCII). Transcobalamin II is now known as transcobalamin. This cobalamin bound to TCII is called holotranscobalamin (holoTC). Most of the holoTC is taken up by the liver where it is degraded and stored, with a portion of the holoTC-TCII complex being secreted into the bloodstream where it can be delivered to various peripheral tissues [3]. HoloTC is metabolically active and rapidly delivered to peripheral tissues, whereas cobalamin bound to HC is inactive. While most cobalamin absorption occurs via IF-mediated mechanisms, a small fraction is absorbed through passive diffusion [4]. In PA, autoimmune destruction of IF-producing PCs, driven by parietal cell antibodies (PCAs), leads to CD (Figure 1). Cobalamin is essential for hematopoiesis and neuronal myelination. Once cobalamin is taken up by peripheral tissue, it is converted into methylcobalamin (MeCbl) and adenosylcobalamin (AdoCbl). MeCbl is a cofactor for methionine synthase, which catalyzes the conversion of homocysteine (Hcy) to methionine. AdoCbl is a cofactor for methylmalonyl CoA mutase, which converts methylmalonyl CoA to succinyl CoA. Thus, CD leads to elevated Hcy and methylmalonic acid (MMA) (Figure 2), both of which are considered neurotoxic and may contribute to demyelination and neuronal death [5].

## 3. Challenges in the Diagnosis of Pernicious Anemia

### 3.1. Challenges Due to Diverse Clinical Presentation

The clinical manifestation of PA is heterogenous and ranges from asymptomatic to severe presentations. The typical presentation of PA may include macrocytic anemia and features of anemia such as fatigue and pallor. In more severe circumstances, patients may experience neurological signs and symptoms which may take several years of development. Thus, early recognition of these patients is paramount. However, the clinical diagnosis may be difficult in cases where neurological symptoms are the first presenting feature of PA without overt clinical signs of anemia or macrocytosis. For instance, Lindenbaum and colleagues found that 28% of 141 patients with CD experienced neuropsychiatric symptoms without anemia or macrocytosis [6]. Interestingly, the severity of anemia does not necessarily correlate with the severity of neurological manifestations. In some cases, the degree of anemia has been reported to be inversely related to neurological severity, with patients presenting with significant neuropsychiatric abnormalities despite mild or absent hematologic findings [7]. In neuropsychiatric patients, diagnosis is further complicated by overlapping symptoms between the primary disorder and CD. Although many of the neurological and psychiatric manifestations described below have been reported across multiple causes of CD, recognition is particularly important in PA because the underlying autoimmune malabsorptive process often remains undiagnosed for prolonged periods.

#### 3.1.1. Neurological Presentation and Pitfalls

The initial neurological manifestation of PA is demyelination, which can progress to axonal degeneration and neuronal death if untreated [8]. This may occur due to the neurotoxic buildup of Hcy and MMA, leading to spinal cord demyelination. Paresthesias are the most commonly reported neurological manifestation of PA and are often the initial presentation. Among 103 patients, the most commonly reported symptom was fatigue (55%), which was followed by sensation disturbance in the limbs (32%) [9]. The most well-characterized myelopathy associated with PA is subacute combined degeneration (SCD) of the dorsal and lateral spinal cord, causing impaired vibration and position sense. However, distinguishing PA-related neuropathy from other neurological disorders, such as polyneuropathy, is challenging, as it may be unclear whether CD is the primary cause or a coexisting condition [10].

The differential diagnoses for SCD should also be considered for other conditions that affect the dorsal and lateral columns of the spinal cord due to similar clinical and neuroimaging results. This includes nutritional deficiencies (e.g., copper, vitamin E), drug-induced etiologies (e.g., methotrexate), infectious causes (e.g., syphilis), and demyelinating disorders (e.g., multiple sclerosis, transverse myelitis) [11].

One study found that there were clinical differences in polyneuropathy patients with CD and those with sufficient cobalamin levels. The authors found that those with CD were 22 times more likely to have upper and lower extremity involvement. However, no conclusions can be drawn as these neurological features may not be distinct enough to attribute to CD due to diagnostic limitations [12]. However, trials featuring a larger patient population are required to further explore the clinical differences between PA and non-PA patients with neurological manifestations. Patients with neuropsychiatric disease, such as those with encephalopathy, may mask a PA-associated myelopathy [13].

PA may also involve damage to the cranial nerves, particularly the optic and olfactory nerves. Optic neuropathy is present in less than 1% of patients with CD. The most common manifestations involve atrophy of the optic nerve without neuronal inflammation [10]. Post-mortem fundoscopy results of PA patients demonstrated optic nerve damage [14]. This suggests the presence of subclinical optic neuropathy.

Neurological symptoms due to CD have also been observed in pediatric patients, including hypotonia, tremors, seizures, and irritability [15]. If untreated, this can lead to developmental delays. In some cases, infants present with failure to thrive due to maternal CD. However, most studies focus on adults, particularly the elderly, highlighting a gap in the literature on pediatric PA.

#### 3.1.2. Cognitive Presentation and Pitfalls

PA has also been associated with cognitive symptoms. Cognitive impairment associated with PA can present with subtle and nonspecific features, including impaired attention, slowed processing speed, memory difficulties, executive dysfunction, and behavioral changes. These manifestations may occur before the development of classic hematologic abnormalities, making recognition particularly challenging.

A major diagnostic pitfall is the attribution of cognitive decline to primary cognitive disorders without consideration of reversible metabolic causes. Seage and colleagues noted that, among 103 patients, 20% had cognitive symptoms, though confirmatory cognitive assessments were lacking [9]. These neurodegenerative changes associated with CD may be related to synaptic dysfunction similar to pathological mechanisms observed in Alzheimer’s disease. Preclinical studies suggest contributing factors include deficits in early-phase long-term potentiation, reduced GluR1-containing alpha-amino-3-hydroxy-5-methyl-4-isoxazolepropionic acid (AMPA) receptor expression, and N-methyl-D-aspartate (NMDA) receptor dysfunction [16]. Furthermore, some studies have found that patients with lower cobalamin levels may be associated with an accelerated rate of global brain atrophy and faster decline in executive function [17]. Recent large-scale data highlight that maintaining adequate vitamin B12 levels—especially through early, long-term intervention—is crucial for protecting cognitive health and lowering dementia risk as populations age [18]. Hyperhomocysteinemia has also been associated with cognitive decline, providing a possible link between biochemical abnormalities in CD and neurologic manifestations [19]. Importantly, cognitive symptoms may improve following cobalamin replacement, although recovery is variable and depends on the duration and severity of neurologic involvement [20]. Therefore, recognizing PA as a potentially reversible contributor to cognitive decline is essential, as delayed diagnosis and treatment may allow progression of neurologic injury despite later correction of biochemical abnormalities.

#### 3.1.3. Psychiatric Presentation and Pitfalls

PA has been associated with psychiatric manifestations such as depression, psychosis, and delirium, although they are less common (14%) [9]. A meta-analysis of 1934 cobalamin-deficient patients found a significant association between CD and geriatric depression in women [21]. One proposed mechanism is impaired monoamine neurotransmitter production [22]. In some cases, this can occur without other neurological features. Patients who present with only psychiatric symptoms are likely to experience diagnostic delays. This can result in progression to severe presentations such as catatonia [23]. CD, including PA, should be considered in the differential diagnoses for patients with treatment-resistant psychiatric disorders.

Hu and authors demonstrated a causal relationship between cobalamin levels and risk of anxiety disorders and bipolar disorders [24]. However, more research is required to fully explore psychiatric disorders with laboratory markers of PA.

At present, cobalamin levels are often obtained as part of workup for cognitive disorders; however, this is not the case for psychiatric disorders such as major depressive disorders. One retrospective study of an older adult psychiatric inpatient cohort suggested that it may be justified to screen for CD regardless of cognitive disorder symptoms based on clinical benefit and cost effectiveness [25].

Psychiatric manifestations, particularly hallucinations, represent another critical diagnostic challenge where PA can be misidentified as a primary psychotic or schizoaffective disorder. A recent systematic review of 50 case studies highlighted that auditory and visual hallucinations served as the primary presentation of CD, appearing before other clinical signs and resolving rapidly with treatment (75% showing full resolution within two months). Of note, only 32% of these hallucinatory cases presented with comorbid PA, reinforcing the reality that severe perceptual distortions often manifest via entirely divergent pathways in the complete absence of typical hematological markers [26].

### 3.2. Challenges Due to Diagnostic Tools

In addition to the clinical diagnosis of PA, there are also criteria which rely on laboratory and other clinical features (Table 1).

Due to subclinical and atypical presentations of PA, clinicians often rely on identifying low cobalamin levels. There are various challenges in diagnosing PA due to the plethora of diagnostic assays available. The measurement of cobalamin is widely used in clinical practice. However, there are many limitations to its use, including false negatives and false positives affected by non-cobalamin factors. HoloTC levels are also a promising diagnostic tool. Functional measurements of cobalamin levels are Hcy and MMA. Each method has advantages and disadvantages, as summarized in Table 2a,b. This discussion will focus on the limitations of each diagnostic method.

The diagnosis of PA is challenging due to its subclinical and atypical presentations, as well as the variety of available diagnostic assays. Clinicians often rely on low serum cobalamin levels, though this test has limitations. This includes spurious results affected by various physiological and pathological states. HoloTC is a promising alternative marker, while functional assays such as Hcy and MMA provide additional diagnostic insight. Given that PA is caused by IFAs and that the autoimmune CAG is caused by anti-PC antibodies, serologic testing of both antibodies can aid in confirmation. This discussion will examine the limitations of each diagnostic method.

#### 3.2.1. Markers of Anemia

PA classically presents with laboratory findings of macrocytic anemia. Macrocytosis is often the initial finding (Table 2a,b). However, these markers of anemia, including hemoglobin and mean corpuscular volume, may be absent. Some patients may have macrocytosis without anemia, and others may present without macrocytosis or anemia. For instance, in 19% of neuropsychiatric patients with CD, anemia and macrocytosis were absent [6]. Furthermore, there should also be concomitant measurement of iron studies in order to determine if iron deficiency is present. Changes may also be observed on the peripheral blood smear (PBS) as well as on bone marrow (BM) biopsy. PBS may reveal macro-ovalocytes and neutrophil hypersegmentation, and BM biopsy can show characteristic changes. However, these findings are typically seen in more advanced cases of anemia and may be absent in patients presenting solely with neuropsychiatric symptoms.

#### 3.2.2. Cobalamin Assays

##### Total Serum Cobalamin Level

Historically, the Schilling test was used to assess cobalamin absorption by measuring urinary excretion of radiolabeled vitamin B12. However, this method is no longer used in modern practice. The most widespread method of evaluating CD is serum cobalamin measurement (Table 2a,b). This test (low cobalamin level) is highly sensitive (95–97%) in appropriate clinical settings but lacks specificity [28].

Cutoff values for serum CD vary across studies, with only 37% of authors using identical thresholds [29]. A commonly used definition of deficiency is a serum cobalamin level <200 pg/mL, while levels between 200–300 pg/mL are often considered borderline and >300 pg/mL sufficient. However, no universal consensus exists, and interpretation should consider clinical context and additional biomarkers. Using the traditional 200 pg/mL cutoff, only 5–12% of the population is classified as deficient, whereas raising it to 300 pg/mL increases the prevalence to 34–50%, potentially leading to overdiagnosis. Notably, some patients with high–normal cobalamin levels (>350 pg/mL) still exhibit elevated MMA and Hcy, highlighting the limitations of serum cobalamin as a standalone diagnostic marker [28].

Serum cobalamin levels can be influenced by various physiological and pathological factors, including age, ethnicity, and underlying diseases. Patients with PA may present with spuriously normal or elevated cobalamin levels [30]. Conversely, low results may be observed in folate deficiency, multiple myeloma, Human immunodeficiency virus (HIV) infection, as well as in pregnancy [22,28,31]. In contrast, conditions associated with increased cobalamin-binding proteins, including myeloproliferative diseases, leukemias, lymphomas, and liver disease, may result in normal or elevated serum cobalamin levels despite underlying deficiency [22,28]. One study found that Black patients and children tend to have higher cobalamin concentrations, possibly due to reference intervals being derived primarily from white adult populations [42]. Thus, implementing age- and ethnicity-specific reference ranges may improve diagnostic accuracy in diverse populations. Furthermore, different immunoassays yield varying reference intervals for serum cobalamin. However, these methods are not interchangeable. A study by Andersen and colleagues highlighted that a patient classified as deficient by one assay may be considered sufficient by another, underscoring the need for standardized diagnostic approaches [43]. For instance, competitive-binding luminescence assay (CBLA) has gained popularity over microbiologic and radioisotope-dilution assays, but it is also prone to diagnostic errors [44]. This underscores the need for standardized diagnostic methods that account for assay variability, ensuring more reliable and consistent identification of B12 deficiency. Serum cobalamin may also be falsely normal or high in 22–35% of patients with PA due to interaction of IFA with IF reagent using the current CBLA [30,44]. The highest level of falsely elevated serum cobalamin due to interference by IFA documented in the literature was 7580 pg/mL in a patient with PA-related SCD of the spinal cord [45].

Very occasionally, CD can be masked by a condition termed macro-B12. In this clinical scenario, the patient has CD; however, the serum cobalamin is elevated as a consequence of complex formation of cobalamin binding proteins with immunoglobulins (macro-B12). To get the actual serum value of cobalamin level, the macro-proteins have to be precipitated with polyethylene glycol (PEG). After PEG precipitation, the spuriously elevated cobalamin level comes down to its actual level. According to 2 studies, the prevalence of macro-B12 ranged from 8% to 25% of samples with persistently high serum cobalamin levels [46,47,48].

##### Holotranscobalamin (HoloTC) Levels

HoloTC represents another method for assessing cobalamin levels. Although no universally accepted cutoff exists, holoTC may improve diagnostic assessment, particularly in patients with borderline serum cobalamin levels. Cutoff values ranged between 20 and 50 pmol/L in some studies [29]. Earlier studies have suggested that holoTC concentrations <35 pmol/L in combination with an elevated MMA level (>271 nmol/L) may support a diagnosis of CD. More recent guidance recommends interpreting holoTC values based on clinical context, with concentrations <25 pmol/L suggestive of deficiency and 25–70 pmol/L considered indeterminate, warranting additional evaluation with functional biomarkers such as MMA [32,33]. However, assay-specific reference intervals vary substantially across laboratories, limiting standardization and potentially leading to different interpretations of borderline results (Table 2a,b). While some studies suggest holoTC is superior to serum cobalamin, limitations include indeterminate results and assay variability [2,29,30,31]. Interestingly, in one comparative study evaluating 11,833 samples, HoloTC seemed to be the preferred first-line marker for detecting subclinical vitamin B12 deficiency in women 50 years and older [49]. Some authors also suggest that holoTC may be a more reliable biomarker than serum cobalamin for early diagnosis of vitamin B12 deficiency [50]. Low holoTC levels may reflect either impaired intestinal absorption of cobalamin or inadequate delivery of cobalamin to peripheral tissues. Because holoTC measures the circulating biologically active fraction of cobalamin, it cannot reliably distinguish between these mechanisms [28,51]. Transient factors, such as reduced cobalamin intake or medication-induced malabsorption, may cause temporary declines in holoTC without leading to true deficiency. Additionally, most clinical data on holoTC focus on subclinical or nonprogressive cases, whereas PA typically presents with more severe and progressive symptoms [28]. Certain conditions affecting the kidney and liver can also result in falsely elevated holoTC levels [52]. Furthermore, some researchers have proposed measuring the ratio of holoTC to total transcobalamin (holoTC:total TC) as a potential alternative diagnostic method [3]. However, this approach is not yet clinically available at most centers.

### 3.3. Functional Measures of Cobalamin Deficiency

Functional measures of CD are most commonly characterized by elevated Hcy and elevated MMA. This differentiates it from folate deficiency, which is characterized by normal MMA levels. These tests are often utilized when cobalamin levels are unequivocal.

#### 3.3.1. Homocysteine (Hcy)

Hcy is a functional marker of CD and may become elevated before hematologic manifestations develop. However, because numerous conditions influence Hcy concentrations, an elevated level should be interpreted in conjunction with serum cobalamin, MMA, and the clinical presentation (Table 2a,b). Spuriously elevated Hcy levels may be observed in renal impairment, volume contraction, vitamin B6 deficiency, or hypothyroidism [53]. Hcy levels may exhibit less specificity compared to MMA as they are frequently elevated in folate deficiency. Some medications may also result in falsely low levels. For instance, Hcy levels may be inversely related to exogenous estrogen and tamoxifen [19,54].

Two main methods are used to measure Hcy levels: chromatographic methods and immunoenzymatic methods. Chromatographic methods are more labor-intensive and require more technical skill, whereas immunoenzymatic methods are simpler and more cost-effective. Although there have been similar results when comparing the two assays, there is also considerable variation in results [55]. Thus, there is a need for standardization of Hcy assays.

#### 3.3.2. Methylmalonic Acid (MMA)

MMA is a highly sensitive marker for CD in overt PA, with elevations seen in over 95% of cases (Table 2a,b) [28]. However, its specificity remains uncertain, as various factors can influence its levels. MMA levels may be elevated out of proportion to cobalamin status in patients with renal failure, old age, volume depletion, as well as possible bacterial overgrowth in the intestines [56]. Falsely low results have been observed in those taking antibiotics [6]. However, these only constitute a minority of cases. MMA levels are not static, with some cases showing progression or decline over time [57]. A review of 44 studies found cutoff values ranging from 210 to 470 nmol/L [29]. However, in the clinical setting, the most commonly used cutoff value for MMA is 270 nmol/L [28]. Longitudinal studies show that MMA fluctuates over time, with only 16% of cases progressing, while 44% experience spontaneous decline, highlighting its limitations as a definitive diagnostic tool. Another way to diagnose CD is reduction in serum MMA by cobalamin supplementation. Herrmann et al. reported that a reduction in MMA ≥ 200 nmol/L from the baseline after cobalamin injections confirms the diagnosis of CD in patients with renal disorders [32]. Hara et al. also reported a case of CD presenting with SCD of the spinal cord and normal serum MMA. The diagnosis of CD was confirmed by lowering of serum MMA by cobalamin administration and improvement of the neurological symptoms [58].

### 3.4. Autoantibodies of Pernicious Anemia

PA can also be confirmed with autoantibody status against IFs and PCs (Table 2a,b). As previously described, IF is a key protein which binds to cobalamin in order to facilitate its delivery and absorption to the terminal ileum. In PA, IF deficiency is often due to autoantibodies that either block the cobalamin binding site, preventing cobalamin–IF complex formation (type 1), or inhibit IF binding to the ileal mucosa (type 2). Type 1 IFAs are present in approximately 70% of patients, while type 2 IFA occurs in 35–40%, often coexisting with type 1 autoantibodies [40,59]. The specificity of IFA is almost 100% with a variable sensitivity (50–70%). Other authors noted a sensitivity varying around 50–70% [38]. False-positive IFA can be seen if the IFA sample is drawn after cobalamin injections. Therefore, it is important to sample IFA before cobalamin administration [60].

PCs are responsible for secreting gastric acid and IF. PCA is present in 80–90% of PA patients, particularly in earlier stages [61]. However, their prevalence decreases to 55% in advanced disease [60]. Testing for PCA via indirect immunofluorescence is more sensitive but less specific. Furthermore, IFAs have been associated with 60% of PA cases. Lahner and Annibale evaluated the diagnostic accuracy of IFA and PCA using ELISA. They determined that IFA had a sensitivity of 37% and specificity of 100%, while PCA had a sensitivity of 81.5% and specificity of 90.3% [62]. The sensitivity of IFA increases to 80% as PA progresses [63]. When both autoantibodies were combined, the sensitivity was 73% and specificity was 100%, demonstrating improved performance [62]. Although these tests are highly specific, PCA may also be positive in other autoimmune diseases such as autoimmune thyroid disease. While PCA is a highly sensitive screening tool, other studies have noted low specificity [39]. Low-titer positive results may occur in healthy older individuals and in patients with other autoimmune disorders, including autoimmune thyroid disease and type 1 diabetes, thereby limiting specificity. In contrast, higher PCA titers appear to correlate more strongly with autoimmune gastritis and PA, with some studies suggesting that values exceeding 100 U/mL may have very high specificity for advanced corpus atrophy [40]. Therefore, quantitative PCA levels may provide additional diagnostic information beyond a simple positive or negative result.

### 3.5. Other Diagnostic Measures

#### 3.5.1. Evidence of Gastric Mucosal Atrophy and Gastric Function

The diagnosis of PA is closely associated with autoimmune CAG. This can be evaluated through biochemical markers such as elevated fasting serum gastrin and low pepsinogen I. These markers are present in 80–90% of PA patients but lack specificity. Thus, their clinical utility may be limited to equivocal cases. The diagnosis of PA is also supported by demonstrating histopathology of CAG. Classical findings include atrophy of the corpus, with sparing of the antrum, as well as hyperplasia of the enterochromaffin-like cells as a result of hypochlorhydria. However, antral involvement does not exclude PA diagnosis [62]. Furthermore, endoscopy is limited by low sensitivity and specificity. In earlier states of autoimmune CAG, there may also be minimal macroscopic changes.

#### 3.5.2. Neuroimaging

Imaging is not typically used to diagnose PA but can aid in cases with neurological symptoms and no overt anemia. While magnetic resonance imaging (MRI) is not a primary diagnostic tool, it plays an important role in ruling out structural mimics and investigation of alternative diagnoses. In the case of SCD, MRI may help to confirm the diagnosis [64].

Spinal MRI helps detect abnormalities, with early findings such as swelling of the myelin sheath; however, neuroimaging is more likely to capture advanced cases such as SCD of the spinal cord (Table 2a,b). The typical findings for SCD are V-shaped T2 hyperintense signals affecting the cervical cord, and bilateral nodular shapes in the thoracic cord, with or without demyelination [65]. This typically starts in the upper thoracic region, and may progress to involve the cervical tract. Although SCD has characteristic findings on MRI, these features may also be variable, which can lead to diagnostic delays [11]. Some changes resolve after treatment, though advanced cases may have persistent abnormalities [5]. Furthermore, diagnostic challenges may arise from conditions that mimic SCD findings in MRI, including multiple sclerosis and amyotrophic lateral sclerosis [11].

Brain MRI in cognitively impaired patients may reveal periventricular white matter hyperintensities (Table 2a,b) [22]. However, many structural changes may be nonspecific, making it challenging to attribute them solely to PA. Additionally, advanced cases may show brain atrophy, though this is not unique to PA and can be seen in other neurodegenerative conditions. Although less common, brain MRI in SCD may also demonstrate patchy T2-weighted and fluid-attenuated inversion recovery (FLAIR) hyperintensities [11]. Therefore, while imaging can provide supportive evidence, it should be interpreted in conjunction with clinical and laboratory findings.

On FLAIR and T2-weighted sequences, patients with severe neurological involvement may demonstrate extensive white matter hyperintensities extending into the deep cerebral white matter. These abnormalities are thought to reflect demyelination and white matter injury associated with CD [41]. In some patients, cerebral atrophy may also be observed. Importantly, some reports have demonstrated partial or complete resolution of white matter abnormalities following parenteral cobalamin replacement, highlighting the potential reversibility of PA-associated neuroimaging findings [22]. Recognition of this radiological reversibility may help distinguish PA-related neurological disease from progressive neurodegenerative disorders and underscores the importance of early diagnosis and treatment.

## 4. Diagnostic Approach to Pernicious Anemia

The diagnostic approach to PA requires a thorough clinical evaluation, including a detailed history, particularly family and autoimmune history, and physical examination, while maintaining broad differential diagnoses. PA commonly coexists with other autoimmune disorders such as type 1 diabetes mellitus, Graves’ disease, Addison’s disease, and vitiligo. Thus, patients presenting with neuropsychiatric symptoms of unclear etiology should be investigated for these conditions. Recently, the Delphi Expert Consensus agreed that recognition of clinical symptoms should receive the highest priority in establishing diagnosis, with laboratory work acting as supportive evidence [64].

In 2024, the National Institute for Health and Care Excellence (NICE) published updated guidance for the diagnosis and management of vitamin B12 deficiency. NICE recommends initial testing with total serum vitamin B12 or active vitamin B12 (holotranscobalamin) in patients with relevant symptoms or signs and risk factors for deficiency. Importantly, the guideline recognizes that clinical manifestations may include neurological symptoms, cognitive changes, and paresthesias, in addition to classical hematological findings. MMA testing can be considered when initial results are indeterminate or when clinical suspicion remains despite inconclusive laboratory findings [37]. The guideline further emphasizes that neurological manifestations may occur without typical hematological abnormalities and that treatment should not be delayed when clinically significant deficiency is suspected. In patients with suspected autoimmune gastritis as an underlying cause, IFA testing is recommended; however, a negative result does not exclude the diagnosis due to limited sensitivity [66].

Despite these recommendations, Thain and colleagues highlight ongoing challenges in the recognition and diagnosis of PA. They emphasize that PA represents an autoimmune disorder causing impaired vitamin B12 absorption rather than simply a cause of anemia, and therefore the absence of anemia or macrocytosis should not exclude the diagnosis. Furthermore, current biomarkers have limitations, and no single test has sufficient sensitivity and specificity to definitively confirm or exclude PA [37].

**Figure 3:**
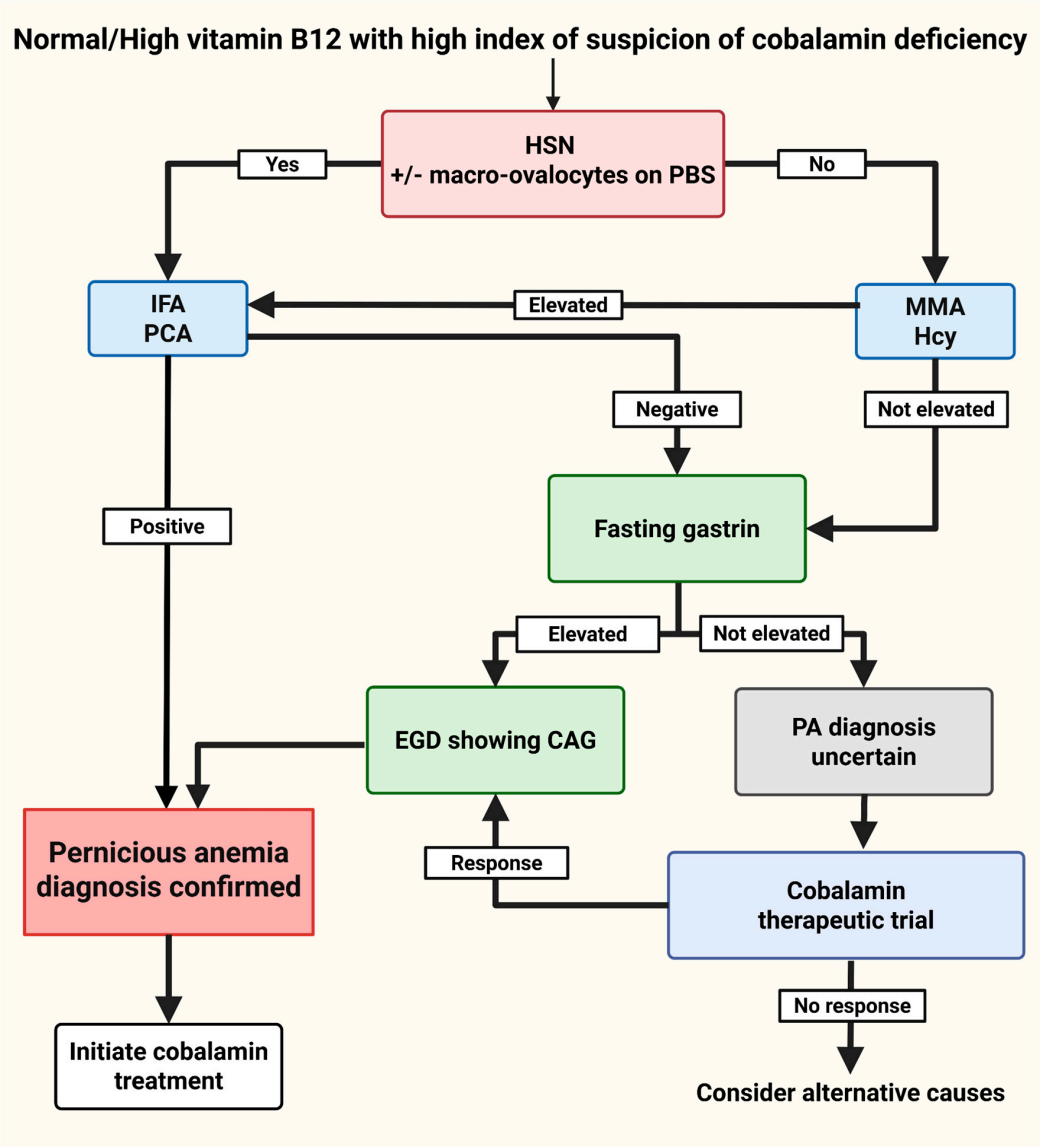
**Diagnostic algorithm for pernicious anemia in patients with suspected cobalamin deficiency despite normal or elevated serum vitamin B12 levels.** Clinical evaluation begins with a peripheral blood smear (PBS); the presence of hypersegmented neutrophils (HSNs) with or without macro-ovalocytes prompts immediate intrinsic factor antibody (IFA) and parietal cell antibody (PCA) testing. If PBS findings are absent, methylmalonic acid (MMA) and homocysteine (Hcy) levels are assessed. Elevated metabolites proceed to IFA/PCA testing, while normal levels direct to fasting gastrin measurement. Positive autoantibody results confirm PA, indicating the initiation of cobalamin therapy. Negative autoantibody panels require fasting gastrin evaluation; elevated gastrin prompts an esophagogastroduodenoscopy (EGD) to confirm chronic atrophic gastritis (CAG) and PA. Normal gastrin levels indicate an uncertain diagnosis, necessitating a cobalamin therapeutic trial. A positive clinical response warrants subsequent EGD evaluation for CAG, whereas no response requires investigation of alternative causes. Created in BioRender. Myat, Y. (2026) https://BioRender.com/1zwhflt, accessed 20 June 2026. **Abbreviations:** HSN, hypersegmented neutrophils; MMA, methylmalonic acid; Hcy, homocysteine; IFA, intrinsic factor antibodies; PCA, parietal cell antibodies; PA, pernicious anemia; EGD, esophagogastroduodenoscopy; CAG, chronic atrophic gastritis; PBS, peripheral blood smear.

Discordant biomarker patterns can further complicate the diagnosis of PA. Patients with neurological manifestations suggestive of PA may have normal serum cobalamin levels, as total serum cobalamin may not accurately reflect intracellular availability. In patients with high clinical suspicion despite a normal cobalamin level, assessment of MMA and Hcy can help identify functional CD [67]. An elevated MMA level with a normal serum cobalamin level should prompt further evaluation rather than excluding the diagnosis. Additional testing for IFAs, PCAs, and evidence of autoimmune atrophic gastritis may help establish the underlying etiology. Ultimately, diagnosis requires integration of clinical findings with complementary biochemical and serologic testing [68]. A proposed diagnostic algorithm for the evaluation of suspected pernicious anemia in patients with normal or elevated serum cobalamin levels is presented in Figure 3.

Some studies suggest that combining serum cobalamin, holoTC, MMA, and Hcy into a single diagnostic model may improve accuracy, though this approach remains unvalidated in clinical practice [49,69]. In challenging cases, fasting serum gastrin, pepsinogen I, and, if necessary, endoscopy with gastric biopsy may provide additional diagnostic clarity [62]. Empirical cobalamin supplementation may be considered in patients with high clinical suspicion, and particularly those with refractory neuropsychiatric symptoms. Additional testing, including iron studies and folate levels, is essential to rule out concurrent deficiencies. Despite ongoing research, clinical trials remain limited, and current studies are underpowered to establish definitive diagnostic protocols.

Given the challenges in diagnosing PA, a prompt and targeted management approach is essential to prevent irreversible neurological complications and optimize patient outcomes. Several cases in the literature demonstrate that cobalamin supplementation results in improvement of neuropsychiatric symptoms [6,12]. The parenteral route is preferred, particularly if neurological features are already present. Some have suggested that patients with a severe neurological presentation should be treated with high-dose intramuscular treatment on a daily basis for one week, then weekly until clinical improvement [70]. Some treated patients who discontinued maintenance doses of cobalamin had recurrent clinical episodes. In a notable retrospective cohort, 46 out of the 189 patients experienced neurological symptoms or signs that did not respond to therapy. This suggests that the neurological symptoms may be attributed to causes other than CD [14]. Ata and colleagues recommend that in patients with non-ophthalmologic symptoms, optic nerve damage should be ruled out with fundoscopy [10].

This review has several limitations. The available literature on PA in neuropsychiatric patients is largely based on observational studies and case series. In addition, significant heterogeneity exists in diagnostic criteria and biomarker thresholds for CD, including serum cobalamin, MMA, Hcy, and holoTC, which limits direct comparison across studies. Furthermore, data specifically isolating neuropsychiatric presentations of PA remain limited, as many studies group all causes of CD together or include mixed neurological populations. Finally, as a narrative review, the selection of included studies may be subject to inherent publication and selection bias.

## 5. Conclusions

Compared with patients who present with the classic hematologic manifestations of PA, individuals with predominant neuropsychiatric symptoms pose significantly greater diagnostic challenges. Many individuals with early-stage PA remain asymptomatic, while those with classic presentations are readily identified by standard hematological parameters such as macrocytosis and anemia. However, these hallmark hematological findings are frequently absent in patients presenting with neuropsychiatric symptoms. Consequently, these patients are often misdiagnosed due to overlapping clinical features with primary psychiatric and neurological disorders. Delayed diagnosis in this subset carries a risk of permanent neurological injury, underscoring the importance of maintaining a high index of clinical suspicion in affected patients.

Beyond clinical presentation, diagnosing PA in neuropsychiatric settings introduces practical and interpretive challenges not typically encountered in non-neuropsychiatric cohorts. Reliance on traditional hematological markers alone is insufficient, as neurological symptoms may precede anemia or macrocytosis. Furthermore, diagnostic methods for PA lack standardization, with variability in biomarker thresholds and limitations in commonly used assays. Overall, this review highlights that diagnosing PA in neuropsychiatric patients requires a more comprehensive and integrated approach. Combining clinical awareness with careful interpretation of multiple diagnostic biomarkers is essential, as no single test is sufficient in isolation. Emphasizing early recognition in this subgroup may reduce the risk of irreversible neurological damage and improve long-term outcomes for patients with PA.

## Figures and Tables

**Table 1:** Pernicious anemia manifestations (references: [1,27]).

Hematological Manifestations	Gastrointestinal Manifestations	Neuropsychitaric Manifestations
Anemic Presentation: Profound fatigue, generalized lethargy, weakness, and exertional dyspnea Pallor (with a characteristic pale-yellow hue from mild concurrent hemolysis) Laboratory findings: Decreased hemoglobin (men: <13 g/dL; women: <12 g/dL) Increased mean corpuscular volume Macro-ovalocytes and hypersegmented neutrophils on peripheral blood smear review Decreased levels of serum cobalamin Intrinsic factor autoantibodies and/or parietal cell autoantibodies	Malabsorptive symptoms Weight loss Abdominal discomfort or bloating Postprandial fullness Gastric mucosal atrophy Chronic autoimmune gastritis Achlorhydria Increased risk of gastric adenocarcinoma and neuroendocrine tumors	Neurological manifestations SCD of the spinal cord (demyelination of posterior and lateral columns) Symmetrical paresthesias (tingling, numbness) starting in lower extremities Loss of vibratory sense and proprioception Ataxic gait and positive Romberg sign Cognitive manifestations Progressive memory impairment and executive dysfunction Reversible dementia syndromes mimicking Alzheimer’s dementia Psychiatric manifestations Severe mood disturbances (treatment-resistant depression, mania) Psychosis, paranoid ideation, visual/auditory hallucinations Anxiety, panic conditions, and personality changes

**Table 2:** (**a**) Advantages and disadvantages/limitations of currently available screening tests in the diagnosis of pernicious anemia. (**b**) Advantages and disadvantages/limitations of currently available supportive and confirmatory tests in the diagnosis of pernicious anemia.

(**a**)						
**Tests**	**Clinical Use**	**Results**	**Sensitivity**	**Specificity**	**Disadvantages/** **Limitations**	**Reference**
CBC and PBS	Initial assessment of hematologic features of PA. Normal findings do not exclude PA, especially patients presenting with neuropsychiatric symptoms	Anemia, other cytopenias, macro-ovalocytes, HSN	≥95% in the right clinical context	Unknown	May mimic other BM disorders such as MDS	[28]
Serum cobalamin	Screening test for suspected PA. Cheap and available at most facilities Borderline or normal results should be interpreted with caution when clinical suspicion is high	<200 pg/mL suggests deficiency; 200–300 pg/mL borderline (no universal consensus)	Variable (95% in the right clinical context)	Unknown	Spurious elevation can be seen in renal failure, macro-B12, hematologic and solid cancers Spuriously high or normal levels due to interference of chemiluminescence assays by the IF antibodies Spuriously low levels in pregnancy, folate deficiency, myeloma, hypothyroidism, and HIV infection	[22,28,29,30,31]
HoloTC	May identify early cobalamin deficiency before serum cobalamin declines	<25 pmol/L suggests deficiency; 25–70 pmol/L considered indeterminate Earlier studies have used <35 pmol/L with elevated MMA as supportive evidence of deficiency	55–87% (variable) Some selected studies may report higher sensitivities	50–96% (variable)	Elevated in renal failure and liver disease Reference ranges differ from one patient population to another depending on cutoff and MMA reference standard Limited availability; not routinely available at many centers	[32,33,34]
(**b**)						
**Tests**	**Clinical Use**	**Results**	**Sensitivity**	**Specificity**	**Disadvantages/** **Limitations**	**Reference**
Hcy	Functional marker of cobalamin deficiency Used in cases where serum cobalamin levels are borderline	>15 μmol/L is a commonly used cutoff. Some studies suggest a lower threshold of >12 μmol/L	High (>90% in many studies)	~50–60% (Variable)	Elevated in renal failure and other large protein meals, pyridoxine deficiency, and hypothyroidism Spuriously low or normal with folate-fortified food, estrogen, and tamoxifen	[35,36,37]
MMA	Functional marker to confirm cobalamin deficiency. Useful in cases where serum cobalamin levels are borderline.	>270 nmol/L is a commonly used cutoff	High (>90% in many studies)	Unknown	Elevated due to reduced clearance in renal failure, which may occur independent of cobalamin status May also be spuriously elevated in old age, plasma volume contraction and intestinal bacterial overgrowth Falsely low with antibiotics	[28]
IFA	Confirmatory test for PA. A positive result strongly supports diagnosis. Not affected by renal dysfunction.	Positive	50–70%	Almost ~100%	Spuriously positive after cobalamin injections	[38]
PCA	Supportive evidence of PA and possible autoimmune gastritis. Most useful when interpreted alongside IFA and other clinical findings. Not affected by renal dysfunction.	Positive	85–90%	Variable (lower specificity due to overlap with auto-immune disorders)	Positive in autoimmune thyroid disease, vitiligo, celiac disease and normal population Variation based on assay	[39]
Upper GI endoscopy with gastric biopsy	Not a primary diagnostic test for PA. Used when serologic and biochemical findings are inconclusive or when confirmation of autoimmune gastritis is required.	Positive for ACAG	100%	100% for ACAG	Invasive, expensive, and labor intensive	[40]
MRI brain/spine	Not a primary diagnostic test for PA. MRI spine may support diagnosis of SCD and for investigation of alternative neurological diagnoses. MRI brain may demonstrate periventricular white matter hyperintensities.	T2 hyperintensity of posterior columns,”inverted V sign”, white matter abnormalities	Not well defined	Not well defined	May be normal in early disease. Findings are not pathognomonic. Similar findings may occur in other conditions including nitrous oxide toxicity, HIV-associated vacuolar myelopathy, and other causes of posterior column dysfunction	[11,41]

Abbreviations: ACAG, autoimmune chronic atrophic gastritis; BM, bone marrow; CBC, complete blood count; Hcy, homocysteine; HIV, human immunodeficiency virus; HoloTC, holotranscobalamin; HSN, hypersegmented neutrophils; IFA, intrinsic factor antibodies; MMA, methylmalonic acid; MRI, magnetic resonance imaging; MDS, myelodysplastic syndromes; PA, pernicious anemia; PBS, peripheral blood smear; PCA, parietal cell antibodies; SCD, subacute combined degeneration.

## Data Availability

No new data were created or analyzed in this study.

## Abbreviations

The following abbreviations are used in this manuscript:

AdoCbl	Adenosylcobalamin
ACAG	Autoimmune chronic atrophic gastritis
AMPA	Alpha-amino-3-hydroxy-5-methyl-4-isoxazolepropionic acid
BM	Bone marrow
CAG	Chronic atrophic gastritis
CBC	Complete blood count
CBLA	Competitive-binding luminescence assay
CD	Cobalamin deficiency
EGD	Esophagogastroduodenoscopy
HC	Haptocorrin
Hcy	Homocysteine
HIV	Human immunodeficiency virus
HoloTC	Holotranscobalamin
HSN	Hypersegmented neutrophils
IF	Intrinsic factor
IFA	Intrinsic factor antibodies
MDS	Myelodysplastic syndromes
MeCbl	Methylcobalamin
MMA	Methylmalonic acid
MRI	Magnetic resonance imaging
NMDA	N-methyl-D-aspartate
PA	Pernicious anemia
PCA	Parietal cell antibodies
PBS	Peripheral blood smear
PCs	Parietal cells
PEG	Polyethylene glycol
SCD	Subacute combined degeneration
TC	Transcobalamin
